# Cementless versus cemented fixation in image-based robotic total knee arthroplasty guided by functional knee positioning principles

**DOI:** 10.1051/sicotj/2025027

**Published:** 2025-05-27

**Authors:** Christos Koutserimpas, Pietro Gregori, Enejd Veizi, Luca Andriollo, Elvire Servien, Cécile Batailler, Sébastien Lustig

**Affiliations:** 1 Orthopaedics Surgery and Sports Medicine Department, FIFA Medical Center of Excellence, Croix-Rousse Hospital, Hospices Civils de Lyon, Lyon University Hospital 103 Grande Rue de la Croix-Rousse 69004 Lyon France; 2 School of Rehabilitation Health Sciences, University of Patras, Campus of University of Patras Rio 26504 Patras Greece; 3 Fondazione Policlinico Universitario Campus Bio-Medico Via Alvaro del Portillo, 200 00128 Roma Italy; 4 Department of Orthopedics and Traumatology, Ankara Yıldırım Beyazıt University, Ankara Bilkent City Hospital Üniversiteler, 1604 06800 Ankara Turkey; 5 Ortopedia e Traumatologia, Fondazione Poliambulanza Via Leonida Bissolati, 57 25124 Brescia Italy; 6 LIBM-EA 7424, Interuniversity Laboratory of Biology of Mobility, Claude Bernard Lyon 1 University 43 Bd du 11 Novembre 1918 69100 Villeurbanne Lyon France; 7 Univ Lyon, Claude Bernard Lyon 1 University, IFSTTAR, LBMC UMR_T9406 25 Avenue François Mitterand 69622 Lyon France

**Keywords:** Functional alignment, Total knee, Cementless, Press fit, Cemented

## Abstract

*Introduction*: Under functional knee positioning (FKPos) principles, residual varus or valgus alignment of the tibia and femur may be maintained, resulting in loading conditions that differ from those observed with mechanical alignment. Consequently, there is a need for evidence regarding implant fixation (cemented or cementless) in this context. This study aimed to evaluate the impact of implant fixation type (cemented versus cementless) on clinical outcomes, complications, and implant survival in robotic-assisted total knee arthroplasty (TKA) guided by FKPos principles. *Methods*: A retrospective comparative analysis of 393 patients who underwent robotic-assisted primary TKA was performed. Patients were divided into two groups: cemented (*n* = 85) and cementless (*n* =276) fixation. Radiographic alignment, functional outcomes using the Knee Society Score (KSS) and Forgotten Joint Score (FJS), complication rates, and implant survival were assessed at a minimum 2-year follow-up. Subgroup analyses based on femoral and tibial fixation types were also conducted. *Results*: Both fixation methods achieved comparable functional outcomes (KSS and FJS) and implant survivorship, with no significant differences in revision rates. Hematomas were significantly more frequent in the cementless group (12.32% vs. 8.24%, *p* = 0.02). Subgroup analyses of femoral and tibial implants showed no significant differences in functional outcomes. *Discussion*: This study is the first to assess the influence of fixation type on outcomes in robotic-assisted TKA performed under FKPos principles. Both cemented and cementless fixation methods are safe and effective.

## Introduction

Total knee arthroplasty (TKA) is a well-established surgical procedure for the management of end-stage knee osteoarthritis, with excellent long-term outcomes [1]. A critical determinant of the success and longevity of the prosthesis is the type of fixation employed, with cemented and cementless fixation being the two primary options [2]. Cemented implants have traditionally been the gold standard due to their predictable outcomes and reliable fixation, particularly in older populations with poorer bone quality [2]. However, advances in biomaterials and implant design have led to a resurgence of interest in cementless fixation, which offers the potential advantages of osseointegration, reduced surgical time, and avoidance of cement-related complications [3]. Studies have demonstrated that cementless implants may achieve comparable or superior fixation in younger, more active patients with good bone quality, although concerns about early loosening and variability in outcomes remain [2, 3]. The growing adoption of image-based assistance and alignment strategies, such as functional alignment, introduces new dimensions to the discussion, as these technologies aim to optimize implant positioning and enhance biomechanical performance, potentially influencing the choice of fixation.

Personalized alignment in TKA optimizes individual joint biomechanics and phenotypes, contrasting with traditional mechanical alignment’s uniform targets [4, 5]. Functional knee positioning (FKPos), also referred to as functional alignment (FA), is a recently introduced personalized alignment strategy for total knee arthroplasty that aims to optimize outcomes by considering each patient’s unique bone anatomy in the coronal and sagittal plane, as well as soft tissue dynamics [6–9]. Unlike traditional alignment methods, which prioritize fixed mechanical principles, FKPos seeks to achieve a three-dimensional alignment tailored to the patient’s specific anatomical features and joint laxity [10, 11]. This is accomplished by balancing mediolateral gaps in knee extension (approximately 10° flexion) and 90° flexion with the aid of advanced robotic systems [12, 13]. These systems have proven to be safe and they enable precise evaluation and real-time adjustment of mediolateral-compartmental gaps, allowing surgeons to customize alignment based on individual anatomic parameters [14–16]. Despite the promise of this approach, evidence regarding the impact of different variables on TKA outcomes under FKPos is still limited. Notably, the influence of implant fixation type, in particular, cemented versus cementless implants, on FKPos outcomes, complications, and revision rates has not yet been systematically evaluated. It should be noted that, according to FKPos principles, residual varus or valgus alignment of the tibia and femur may be maintained, resulting in loading conditions that differ from those observed with mechanical alignment. Consequently, there is a need for evidence regarding implant fixation (cemented or cementless) in this context.

This retrospective comparative study aims to evaluate the effect of implant fixation type (cementless vs. cemented) on clinical outcomes, complications, and revision rates in patients undergoing TKA using the image-based robotic system (Mako, Stryker, Mako Surgical Corp., Fort Lauderdale, FL, USA) guided by the FKPos principles. We hypothesized that there would be no overall difference between the two fixation modalities in terms of complications and clinical outcomes at a minimum 2-year follow-up.

## Materials and methods

This retrospective comparative study utilized data from a prospectively maintained database. The analysis included all adult patients who underwent primary robotic-assisted TKA between March 2021 and January 2023. All procedures were performed with the subvastus approach, without a tourniquet, with intraincisional metaphyseal unicortical femoral pins’ placements as previously described by Koutserimpas et al., all included cases were performed under the principles of FKPos [7, 10, 16, 17]. All patients received either a cruciate-substituting (CS) or a posterior stabilized (PS), fixed-bearing implant (Triathlon, Stryker, MI, US).

Robotic-assisted TKA cases under the principles of mechanical alignment due to soft tissue deficiencies, such as previous fractures, around-the-knee osteotomies, or ligamentous injuries, were excluded. Furthermore, the minimum follow-up period was 2 years; hence, cases with inadequate follow-up periods were excluded. Fixation (cemented or cementless) was determined intraoperatively based on the surgeon’s assessment using trial components.

Patient demographics, including age, sex, and body mass index (BMI), were recorded. Patients were divided into two main groups based on the type of implant fixation used: cementless implants (group A) and cemented implants (group B). In this distinction, both femoral and tibial components had to be cementless or cemented. Then, groups were further subdivided to analyze femoral and tibial implants separately: group A.1: cementless femoral implant, B.1: cemented femoral implant, group A.2: cementless tibial implant, B.2: cemented tibial implant.

A total of 393 cases (241 females; 61.32%) were enrolled in the study with a median age of 70 (Interquartile range (IQR) 64 to 74) and a median BMI of 28.25 kg/m^2^ (IQR 25.2 to 31.64). The median follow-up of the studied population was 30 months (IQR 26 to 37.25).

Radiographic evaluations, in full-length weight-bearing standing X-rays, included coronal alignment measurements, such as the mechanical hip-knee-ankle angle (HKA), the lateral distal femoral angle (LDFA), and the medial proximal tibial angle (MPTA). Data related to alignment and implant positioning were also retrieved from the robotic system.

Clinical outcomes were assessed preoperatively and at the final follow-up using the Knee Society Scores (KSS) for both knee and functional evaluations, as well as the range of motion (ROM). Additionally, the Forgotten Joint Score (FJS) was analyzed at the final follow-up. Complications and revision surgeries were reviewed, with major complications defined as those requiring surgical intervention.

### Statistical analyses

The distribution of data was assessed using the Kolmogorov-Smirnov test. Depending on the presence or absence of normality, either the independent *t*-test or the Mann-Whitney test was applied to compare groups. The chi-square test was employed to analyze differences in complication rates between the two groups. A significance level of *P* < 0.05 was considered statistically significant. Implant survival was analyzed using the Kaplan-Meier method. All statistical analyses were performed using MedCalc software, version 22.021.

## Results

### Overall analysis of cementless versus cemented implants

In 276 cases, both components (femoral and tibial) were implanted without the use of cement (group A), while in 85, both components were cemented (group B). Preoperative characteristics, including demographics, KSS, and radiographic parameters, did not differ significantly between the groups (Table 1). Postoperative evaluations showed comparable functional outcomes, with no significant differences in KSS (knee and function part) and FJS. Radiographic assessments revealed that the cemented group had significantly lower tibial varus (*p* = 0.02) and a higher medial proximal tibial angle (MPTA) (*p* = 0.04). Complication rates, including major complications requiring surgical intervention, were similar between groups (*p* = 0.3) (Table 2).

**Table 1:** Pre- and post-operative data on the prostheses’ groups (groups A and B). Preoperatively, comparative analysis of the demographics, the implant design, the clinical and radiographic evaluation, and the final implants’ positioning. Postoperatively, evaluation of the clinical and radiographic parameters between the two groups. Complications and revisions are also presented. The statistically significant values are depicted in bold.

	Preoperative evaluation		
	Group A (cementless implants) (*N* = 276)	Group B (cemented implants) (*N* = 85)	*P*-value
Demographics			
Age (years)	70 (IQR 64 to 74)	71 (IQR 64 to 74.25)	0.73
BMI (kg/m^2^)	28.25 (IQR 25.43 to 31.65)	28.71 (IQR 24.89 to 31.7)	0.81
Female gender	60.87	63.53	0.66
Implant design			
CR	42.03%	3.53%	**<0.001**
Follow-up			
Period in months	30 (IQR 26 to 37)	30 (IQR 26 to 38)	0.97
Preoperative clinical evaluation			
KSS-knee	67 (IQR 57 to 75.5)	64 (IQR 55 to 76.5)	0.32
KSS-function	70 (IQR 60 to 80)	70 (IQR 55 to 77.25)	0.31
Preoperative radiographic evaluation			
HKA	175° (IQR 172 to 179)	177° (IQR 170 to 184.25)	0.26
LDFA	91° (IQR 90 to 93)	91° (IQR 89 to 93)	0.9
MPTA	87° (IQR 85 to 89)	88° (IQR 84 to 90)	0.38
Robotic alignment evaluation			
Preoperative Mako Alignment (varus)	6° (IQR 2 to 8)	5° (IQR 0 to 9)	0.47
Implant positioning (Mako data)			
Tibial varus	3.5° (IQR 2 to 4.5)	2.25° (IQR 1 to 4)	**0.004**
Tibial ER	0° (IQR 0 to 0)	0° (IQR 0 to 0)	0.13
Tibial posterior slope	1° (IQR 0 to 1)	0.5° (IQR 0 to 1)	0.47
Femoral Valgus	1° (IQR −0.2 to 2)	0.95° (IQR −0.1 to 2)	0.66
Femoral ER	0° (IQR −1.3 to 1.2)	0.3° (IQR −0.95 to 1.2)	0.32
Femoral flexion	7.5° (IQR 5.45 to 9)	7.55° (IQR 6 to 9)	0.65
	Postoperative evaluation		
Postoperative clinical evaluation			
KSS-knee	94 (IQR 90 to 100)	95 (IQR 90 to 100)	0.41
KSS-function	90 (IQR 90 to 94.77)	90 (IQR 88 to 100)	0.89
FJS	84 (IQR 66.25 to 92)	90 (IQR 66 to 100)	0.06
Postoperative radiographic assessment			
HKA	179° (IQR 176 to 180)	179° (IQR 177 to 182)	0.33
LDFA	91° (IQR 89.3 to 92)	90° (IQR 88 to 91)	**0.01**
MPTA	88° (IQR 86.5 to 89)	89° (IQR 87 to 90)	**0.04**
Robotic alignment evaluation			
Postoperative Mako Alignment (varus)	3° (IQR 1 to 5)	1° (IQR 0 to 4)	**0.02**
Complications			
Total complications	12.32% (34 cases)	8.24% (7 cases)	0.3
Major complications	1.45% (3 cases of DAIR and 1 aseptic revision)	3.53% (2 cases of DAIR and 1 case of postoperative fracture)	0.22
Revisions (aseptic loosening)	0.36%	–	0.58
MUA	4.38% (12 cases)	2.35% (2 cases)	0.4
Medial condyle necrosis	–	1.18% (1 case)	0.07
Hematoma	6.16% (17 cases)	–	**0.02**
Infections-DAIR	1.09% (3 cases)	2.35% (2 cases)	0.39
Intra- and post-operative femoral fractures	0.36% (1 intraoperative case)	2.35% (1 intraoperative and 1 postoperative case)	0.08

*N*: number, BMI: body mass index, IQR: Interquartile range, ER: External rotation (Femoral ER with reference to the trans-epicondylar axis, Tibial ER with reference to the Akagi line), KSS: Knee Society Score, HKA: Hip-Knee angle, LDFA: lateral distal femoral angle, MPTA: medial proximal tibial angle.

**Table 2:** Pre- and post-operative data on the femoral prostheses’ subgroups (Groups A.1 and B.1). Preoperatively, comparative analysis of the demographics, the implant design, the preoperative clinical and radiographic evaluation, and the final femoral implant positioning. Postoperatively, evaluation of the clinical, radiographic parameters between the two femoral prostheses’ subgroups. Complications and revisions are also presented. The statistically significant values are depicted in bold.

	Preoperative evaluation		
	Group A.1 (cementless femoral implants) (*N* = 302)	Group B.1 (cemented femoral implants) (*N* = 91)	*P*-value
Demographics			
Age (years)	69 (IQR 63.75 to 74)	71 (IQR 64.5 to 74.5)	0.44
BMI (kg/m^2^)	28.07 (IQR 25.33 to 31.64)	29.03 (IQR 24.91 to 31.83)	0.66
Female gender	60.56%	63.74%	0.59
Implant design			
CR	43.05%	4.4%	**<0.0001**
Preoperative clinical evaluation			
KSS-knee	66.5 (IQR 57 to 76)	60 (IQR 53 to 75)	0.1
KSS-function	70 (IQR 60 to 80)	70 (IQR 53.75 to 75.75)	0.17
Preoperative radiographic evaluation			
HKA	175° (IQR 172 to 179)	177° (IQR 170.25 to 184)	0.21
LDFA	91° (IQR 90 to 93)	91° (IQR 88.25 to 93)	0.76
MPTA	87° (IQR 85 to 89)	88° (IQR 84 to 90)	0.27
Robotic alignment evaluation			
Preoperative Mako alignment (varus)	6° (IQR 2 to 8)	6° (IQR 0.25 to 9)	0.54
Implant positioning (Mako data)			
Femoral valgus	0.9° (IQR −0.23 to 2)	0.6° (IQR −0.1 to 2)	0.71
Femoral ER	0.1° (IQR −1.3 to 1.33)	0.2° (IQR −1 to 1.1)	0.71
Femoral flexion	7.8° (IQR 5.5 to 9)	7.5° (IQR 6 to 9)	0.88
	Postoperative evaluation		
Postoperative clinical evaluation			
KSS-knee	95 (IQR 90 to 100)	95 (IQR 90 to 100)	0.38
KSS-function	90 (IQR 90 to 100)	95 (IQR 88.5 to 100)	0.63
FJS	84 (IQR 67 to 92)	89 (IQR 67 to 98)	0.08
Postoperative radiographic assessment			
HKA	178.85° (IQR 176 to 180)	179° (IQR 177 to 182)	0.44
LDFA	91° (IQR 89 to 92)	90° (IQR 88.3 to 91)	**0.02**
MPTA	88° (IQR 86.5 to 89.3)	89° (IQR 87 to 90)	0.06
Robotic alignment evaluation			
Postoperative Mako alignment (varus)	3° (IQR 1 to 5)	2° (IQR 0 to 4.25)	**0.04**
Complications			
Total complications	11.26% (34 cases)	10.99% (10 cases)	0.94
Major complications	0.99% (3 cases of DAIR)	3.3% (2 cases of DAIR and 1 postoperative fracture)	0.12
Revisions (aseptic loosening)	–	–	0.07
MUA	4.64% (14 cases)	5.49% (5 cases)	0.74
Medial condyle necrosis	–	1.1% (1 case)	0.07
Hematoma	5.3% (16 cases)	–	**0.03**
Infections-DAIR	0.99% (3 cases)	2.2% (2 cases)	0.37
Intra- and Post-operative femoral fractures	0.33% (1 case)	2.2% (2 cases)	0.07

*N*: number, BMI: body mass index, IQR: Interquartile range, ER: External rotation (Femoral ER with reference to the trans- epicondylar axis, Tibial ER with reference to the Akagi line), KSS: Knee Society Score, HKA: Hip-Knee angle, LDFA: lateral distal femoral angle, MPTA: medial proximal tibial angle.

**Figure 1:**
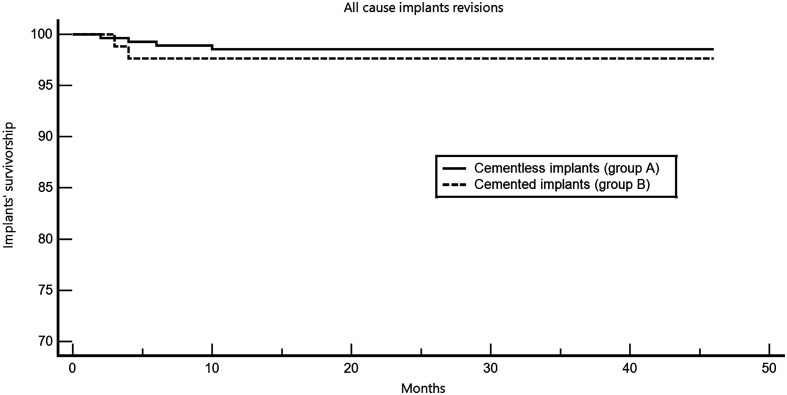
Kaplan-Meyer survival analysis of the two groups, taking into account both infection-related and aseptic causes of revisions. Polyethylene exchange as part of the debridement, antibiotics, and implant retention procedure has been included as implant failure in this analysis. Kaplan Meier survival analysis showed that group B (cemented implants) did not have significantly higher risk of all-cause revision (infections and aseptic loosening) compared to group A (cementless implants) (*p* = 0.57).

The overall complication rate was 12.32% in group A (cementless implants) and 8.24% in group B (cemented implants) (*p* = 0.3). Major complications, defined as those requiring surgical intervention, did not differ significantly between groups. Revision rates for aseptic loosening were low in both groups, with no statistically significant difference. Kaplan Meier survival analysis showed that group B (cemented implants) did not have a significantly higher risk of all-cause revision (infections and mechanical failures) compared to group A (cementless implants) (*p* = 0.57). The all-cause (infection and aseptic loosening) implants’ revision rate was 1.45% in group A and 2.35% in B (Hazard ratio (HR) = 1.74; 95% Confidence interval (CI), 0.26 to 11.52) (Figure 1).

### Femoral implants subanalysis

For the femoral implant subanalysis, 302 cases received cementless femoral implants (group A.1), and 91 cemented femoral implants (group B.1). There were no significant preoperative differences between the groups in terms of demographics or radiographic alignment parameters (Table 3). Postoperative radiographic assessments indicated that the cemented femoral implant group had a significantly higher LDFA (*p* = 0.02). Functional outcomes, measured by KSS and FJS, were comparable between groups. Complication rates were also similar.

**Table 3:** Pre- and post-operative data on the tibial prostheses’ subgroups (Groups A.2 and B.2). Preoperatively, comparative analysis of the demographics, the implant design, the preoperative clinical, and radiographic evaluation and the final femoral implant positioning. Postoperatively, evaluation of the clinical, radiographic parameters between the two tibial prostheses’ subgroups. Complications and revisions are also presented. The statistically significant values are depicted in bold.

	Preoperative evaluation		
	Group A.2 (cementless tibial implants) (*N* = 282)	Group B.2 (cemented tibial implants) (*N* = 111)	*P*-value
Demographics			
Age (years)	70 (IQR 64 to 74)	69 (IQR 63 to 74)	0.48
BMI (kg/m^2^)	28.32 (IQR 25.47 to 31.65)	27.68 (IQR 24.86 to 31.25)	0.27
Female gender	60.99%	62.16%	0.83
Implant design			
CR	41.49%	15.3%	**<0.0001**
Preoperative clinical evaluation			
KSS-knee	66 (IQR 57 to 74.75)	64 (IQR 55.5 to 76)	0.47
KSS-function	70 (IQR 60 to 80)	70 (IQR 57.5 to 76.5)	0.51
Preoperative radiographic evaluation			
HKA	175° (IQR 172 to 179)	176° (IQR 169 to 182)	0.86
LDFA	91° (IQR 91 to 93)	92° (IQR 89 to 93)	0.74
MPTA	87° (IQR 85 to 89)	87° (IQR 84 to 89)	0.97
Robotic alignment evaluation			
Preoperative Mako Alignment (varus)	6° (IQR 2 to 8)	6° (IQR 1.25 to 9)	0.86
Implant positioning (Mako data)			
Tibial varus	3.5° (IQR 2 to 4.5)	2.5° (IQR 1 to 4.5)	0.1
Tibial ER	0° (IQR 0 to 0)	0° (IQR 0 to 0)	0.21
Tibial posterior slope	1° (IQR 0 to 1)	0.5° (IQR 0 to 1)	0.65
Postoperative evaluation			
Postoperative clinical evaluation			
KSS-knee	94.5 (IQR 90 to 100)	95 (IQR 90 to 100)	0.24
KSS-function	90 (IQR 90 to 100)	94 (IQR 88 to 100)	0.73
FJS	84 (IQR 67 to 92)	88 (IQR 67 to 96)	0.08
Postoperative radiographic assessment			
HKA	179° (IQR 176 to 180)	178° (177 to 181)	0.76
LDFA	91° (IQR 89.43 to 92)	90° (IQR 88 to 92)	**0.02**
MPTA	88° (IQR 86.5 to 89)	89° (IQR 87 to 90)	0.05
Robotic alignment evaluation			
Postoperative Mako Alignment (varus)	3 (IQR 1 to 5)	2 (IQR 0 to 5)	0.13
Complications			
Total Complications	11.35% (32 cases)	8.1% (9 cases)	0.34
Major complications	1.42% (3 cases of DAIR and 1 aseptic loosening)	1.8% (2 cases of DAIR)	0.78
Revisions (aseptic loosening)	0.35% (1 case)	–	0.53
MUA	3.9% (11 cases)	5.4% (6 cases)	0.51
Hematoma	6.03% (17 cases)	1.8% (2 cases)	0.08
Infections-DAIR	1.06% (3 cases)	1.8% (2 cases)	0.56

*N*: number, BMI: body mass index, IQR: Interquartile range, ER: External rotation (Femoral ER with reference to the trans-epicondylar axis, Tibial ER with reference to the Akagi line), KSS: Knee Society Score, HKA: Hip-Knee angle, LDFA: lateral distal femoral angle, MPTA: medial proximal tibial angle.

### Tibial implant subanalysis

For the tibial implant subanalysis, 282 cases received cementless tibial implants (group A.2), and 111 received cemented tibial implants (group B.2). Preoperative demographics and radiographic alignment parameters were not significantly different between groups. Postoperatively, the cemented tibial implant group demonstrated significantly lower varus alignment (*p* = 0.02), as determined by data retrieved from the Mako robotic system, and higher MPTA (*p* = 0.05) from the radiographic evaluation. Functional outcomes, as evaluated with KSS and FJS, were similar between groups, and no significant differences in overall complication rates were observed. However, hematomas were more frequent in the cementless tibial implant group (*p* = 0.08), though this finding was not statistically significant.

## Discussion

This study evaluated the impact of implant fixation type, in particular, cementless versus cemented components, on clinical outcomes, complications, and implant survival in patients undergoing robotic-assisted TKA guided by the principles of FKPos. The findings were in concordance with our initial hypothesis and demonstrated that both cemented and cementless fixation methods achieved similar functional outcomes in short-term follow-up, as indicated by comparable KSS and FJS. Implant survivorship was also consistent between the two groups, with no significant differences in revision rates observed.

Complication rates, including overall and major complications requiring surgical intervention, were comparable across the cemented and cementless groups. However, a notable finding was the significantly higher incidence of hematomas in the cementless group (*p* = 0.02). These results reinforce the safety and efficacy of both fixation methods in robotic-assisted TKA while highlighting specific areas where differences may influence clinical decision-making.

The present study’s findings align with existing literature, indicating that both cemented and cementless fixation methods in TKA yield comparable patient-reported outcome measures (PROMs). Studies have demonstrated similar functional outcomes between the two fixation types, with no significant differences in PROMs such as the KSS and FJS [2, 3, 18]. This suggests that both cemented and cementless implants can effectively restore knee function and provide patient satisfaction in the context of robotic-assisted TKA guided by FKPos principles.

This study observed a significantly higher incidence of hematomas in the cementless group (*p* = 0.02), suggesting a potential association between fixation type and postoperative bleeding complications. This finding aligns with existing literature indicating that cementless TKA may lead to increased blood loss compared to cemented TKA [19, 20]. The absence of bone cement in cementless procedures eliminates the tamponade effect provided by cement, potentially resulting in greater bleeding from resected bone surfaces [19]. This highlights the need to consider bleeding risks as part of the decision-making process when selecting fixation types, taking into account that these surgeries will require anticoagulation prevention therapy as well. In cases where a tourniquet is used, which is common practice in many centers, when implanting a cementless prosthesis without the tamponade effect of the cement, the rebound effect after tourniquet release could lead to a higher-than-expected incidence of postoperative hematomas [19]. Effective patient blood management strategies are critical in reducing perioperative bleeding and associated complications, including hematomas [21]. The use of tranexamic acid, meticulous surgical techniques, and optimized intraoperative hemostasis are well-established measures that have been shown to significantly mitigate blood loss in TKA [22]. Adopting such measures not only improves patient outcomes but also reduces the need for transfusions and minimizes the risk of related complications. It is of note that all patients enrolled in this study underwent the same perioperative care, including preoperative administration of 1 g of tranexamic acid. Future research could include a detailed evaluation of the postoperative hemoglobin levels and transfusions between cementless and cemented TKAs.

The differences in radiographic alignment observed in this study can be correlated with the principles of FKPos. As a soft tissue-driven alignment strategy, FKPos focuses on achieving balanced mediolateral gaps in both knee extension and flexion [7, 12, 13, 17]. This approach customizes implant positioning to accommodate each patient’s unique soft tissue envelope, which acts as the knee’s functional “DNA” [11, 23]. Consequently, variations in alignment parameters, such as tibial varus and MPTA, may reflect patient-specific adaptations guided by the intraoperative assessment of soft tissue dynamics [6, 10]. While these differences were statistically significant, it is important to note that they are unlikely to be clinically significant. The HKA was comparable between the cemented and cementless groups, suggesting that overall limb alignment was equally restored regardless of fixation type. This highlights the robustness of the FKPos principles in achieving satisfactory alignment outcomes while accommodating individual anatomical and biomechanical variations.

An observed difference in implant type between the cemented and cementless groups in this study warrants attention. Table 1 shows a higher use of CS implants in the cementless group, while PS implants were more common in the cemented group. This likely reflects clinical preferences to match strategies to patient activity levels, with cementless CS implants often favored for active patients to preserve natural knee kinematics, and cemented PS implants providing enhanced stability for less active patients [24]. Although age was similar between groups, unmeasured factors such as activity levels may have influenced these choices and outcomes. Future studies should explore these associations to further understand their impact on TKA results. Nevertheless, it should be noted that both CS and PS have exhibited similar outcomes in robotic TKA with the FKPos principles [25].

This study has some limitations. The retrospective design and relatively short follow-up period limit the ability to assess long-term outcomes and complications, such as aseptic loosening and late revisions. Further research is needed to explore the long-term implications of fixation type on implant survival and patient outcomes, particularly in the context of robotic-assisted TKA. Prospective studies with longer follow-up durations and larger cohorts are warranted to validate these findings and investigate the interplay between fixation type, alignment strategies, and patient-specific factors, such as activity levels. Nevertheless, it should be noted that this is the first study evaluating the impact of implants’ fixation type on patients undergoing robotic TKA with the principles of FKPos.

In conclusion, this study demonstrated that both cemented and cementless fixation methods achieve comparable functional outcomes and implant survival in robotic-assisted TKA guided by FKPos principles. Hematomas were significantly more frequent in the cementless group, highlighting a potential complication that warrants attention in clinical decision-making. The study is the first to evaluate the impact of implant fixation type within the framework of FKPos and robotic assistance, providing valuable insights into the safety and efficacy of these strategies.

## Data Availability

Data is available upon reasonable request to the corresponding author.
